# Caryophyllene Oxide Induces Ferritinophagy by Regulating the NCOA4/FTH1/LC3 Pathway in Hepatocellular Carcinoma

**DOI:** 10.3389/fphar.2022.930958

**Published:** 2022-07-11

**Authors:** Zhiru Xiu, Yilong Zhu, Jicheng Han, Yaru Li, Xia Yang, Guohua Yang, Gaojie Song, Shanzhi Li, Yue Li, Cheng Cheng, Yiquan Li, Jinbo Fang, Xiao Li, Ningyi Jin

**Affiliations:** ^1^ Academician Workstation of Jilin Province, Changchun University of Chinese Medicine, Changchun, China; ^2^ Medical College, Yanbian University, Yanji, China; ^3^ Changchun Medical College, Changchun, China; ^4^ Changchun Veterinary Research Institute, Chinese Academy of Agricultural Sciences, Changchun, China; ^5^ Jiangsu Co-Innovation Center for Prevention and Control of Important Animal Infectious Diseases and Zoonoses, Yangzhou, China

**Keywords:** ferritinophagy, liver cancer, caryophyllene oxide, NCOA4, Fth1, LC3, ferroptosis

## Abstract

Ferritinophagy is associated with tumor occurrence, development, and therapy effects. Ferritinophagy and ferroptosis are regulated by iron metabolism and are closely connected. LC3 protein is a key protein in autophagy. Following the binding of NCOA4 to FTH1, it links to LC3Ⅱ in lysosomes, a symbol of ferritinophagy. A ferritinophagy’s inducer is likely to open new avenues for anticancer medication research and development. In this study, we discovered that caryophyllene oxide has a substantial inhibitory effect on HCCLM3 and HUH7 cells, by regulating the level of cellular oxidative stress, and the levels of autophagy and iron metabolism in HCCLM3 and HUH7 cells, leading to a ferritinophagy-related phenomenon. Furthermore, the results of T-AOC, DPPH free radical scavenging rate, and hydroxyl radical inhibition indicated that caryophyllene oxide can inhibit cell anti-oxidation. The examination of the ferritinophagy-related process revealed that caryophyllene oxide promotes the production and accumulation of intracellular reactive oxygen species and lipid peroxidation. NCOA4, FTH1, and LC3Ⅱ were found to be targeted regulators of caryophyllene oxide. Caryophyllene oxide regulated NCOA4, LC3 Ⅱ, and FTH1 to promote ferritinophagy. *In vivo*, we discovered that caryophyllene oxide can lower tumor volume, significantly improve NCOA4 and LC3 protein levels in tumor tissue, and raise Fe^2+^ and malondialdehyde levels in serum. The compound can also reduce NRF2, GPX4, HO-1, and FTH1 expression levels. The reduction in the expression levels of NRF2, GPX4, HO-1, and FTH1 by caryophyllene oxide also inhibited GSH and hydroxyl radical’s inhibitory capacities in serum, and promoted iron deposition in tumor tissue resulting in the inhibition of tumor growth. In summary, our study revealed that caryophyllene oxide mostly kills liver cancer cells through ferritinophagy-mediated ferroptosis mechanisms. In conclusion, caryophyllene oxide may be used as a ferritinophagy activator in the field of antitumor drug research and development.

## Introduction

Ferroptosis is a new type of cell death, characterized by the buildup of ROS during the iron-mediated Fenton reaction, resulting in the accumulation of peroxidized lipids containing active iron (Dixon et al., 2012). Ferritinophagy is a crucial mechanism in the regulation of the intracellular iron metabolism (Li et al., 2020). Through ferritinophagy, cells may create unstable iron. Ferritinophagy and ferroptosis are intimately connected with the regulation of the iron metabolism. It has been shown that ferritinophagy is required to trigger ferroptosis (Zhang et al., 2018; Sui et al., 2019). It is a new type of autophagy that is mediated by NCOA4. When an iron-containing ferritin is sequestered inside the autophagosome and ferritin is degraded to produce a large amount of free iron, ferritinophagy occurs, resulting in ferroptosis (Mancias et al., 2014; Tang et al., 2018; Ajoolabady et al., 2021). Ferritinophagy is associated with the pathophysiology of cardiovascular disease and cancer treatment, which has always been a focal area in this research field (Ishimoto et al., 2011; Hao et al., 2017; Ajoolabady et al., 2021). Studies have shown that DpdtC (2,2′-di-pyridylketone dithiocarbamate) suppresses the epithelial–mesenchymal transition of gastric cancer cells through ferritinophagy associated with the ROS-mediated p53/AKT/mTOR pathway (Xu et al., 2020). When investigating the relationship between ferritinophagy and cancer prognosis, researchers found that the expression of NCOA4 in renal cell carcinoma was significantly lower than that in normal renal tissue (Mou et al., 2021). Studies have found that ferroptosis plays an important role in radiotherapy. The ionization radiation of tumor cells results in typical ferroptotic morphological characteristics. Numerous studies have shown that ferroptosis agonists can also enhance the effect of radiotherapy, while ferroptosis inhibitors do the opposite (Lang et al., 2019; Shibata et al., 2019; Lei et al., 2020). As a result, studies into the antitumor mechanism of ferritinophagy might contribute to further understanding of ferroptosis regulation and identify additional potential treatment targets for various diseases. It can also be used in combination with other antitumor means for synergistic treatment, which is of great significance.

Liver cancer is one of the most common primary malignant tumors (Bray et al., 2018; Villanueva, 2019; Kim and Viatour, 2020). The number of new cases is increasing yearly due to its high morbidity and mortality, and its high recurrence, metastasis, and poor prognosis, which seriously endangers human health and life (Kulik and El-Serag, 2019; Rice et al., 2021). Because of the concealment of the development of primary liver cancer, most symptoms appear in the middle and late stages (Shao et al., 2021; Sung et al., 2021). Currently, surgery is one of the most effective strategies for liver cancer treatment (Hirokawa et al., 2020), but there is still a high risk of metastasis and recurrence following surgery (Mavros et al., 2014; Spolverato et al., 2015), and therefore, drug therapy is still an essential way of treating liver cancer (Chen et al., 2020). Sorafenib (Raoul et al., 2019), regorafenib (Nonomiya et al., 2019), lenvatinib (Rimassa et al., 2019), doxorubicin (Outomuro et al., 2007), fluorouracil (Hong et al., 2019; Suwannakot et al., 2021), and other drugs have poor clinical efficacy or serious adverse reactions and side effects in the treatment of advanced liver cancer. Thus, identifying new anti-liver cancer drugs with good efficacy and low side effect has attracted a great deal of interest in the healthcare field. Caryophyllene oxide is a small molecular compound found in a variety of plants (Sanchez-Munoz et al., 2012; Monzote et al., 2018; Ibrahim et al., 2019). One study showed that caryophyllene oxide can improve mitochondrial membrane depolarization and trigger early and late apoptosis in PC-3 cells (Delgado et al., 2021). Caryophyllene oxide has been found to have analgesic and anti-inflammatory effects (Chavan et al., 2010), and it has inhibitory effects on different insect vectors (Monzote et al., 2009; Moreno et al., 2018). Both β-caryophyllene and caryophyllene oxide belong to the natural bicyclic sesquiterpenes (Fidyt et al., 2016). A number of studies have shown that β-caryophyllene has effects on arthritis (Li et al., 2021), rectal cancer (Dahham et al., 2021), and liver cancer (Di Giacomo et al., 2019). However, there is no information on the effect of caryophyllene oxide on HCCLM3 and HUH7 liver cancer cells. Thus, the purpose of this study is to investigate the effect of caryophyllene oxide on liver cancer cells and their related cellular pathways by analyzing the effect of caryophyllene oxide on liver cancer cells and the changes in their specific protein expressions. The results of the study provide a new and improved theoretical framework on the use of caryophyllene oxide in the treatment of liver cancer.

## Materials and Methods

### Reagents and Antibodies

Nuclear factor E2-related factor 2 (Nrf2, Proteintech), heme oxygenase-1 (HO-1, CST), quinone oxidoreductase-1 (NQO1, Proteintech), glutathione peroxidase 4 (Gpx4, Invitrogen), nuclear receptor coactivator 4 h (NCOA4, Sigma-Aldrich), ferritin heavy chain 1 (FTH1, CST), and light chain 3B (LC3Ⅱ, Proteintech) were obtained.

Caryophyllene oxide (CAS:1139-30-6, Sigma-Aldrich, United States), cisplatin and sorafenib (MCE), DMSO (CAS:67-68-5, Sigma-Aldrich, United States), Cell Counting Kit-8 (DOJINDO, Japan), Crystal Violet Staining Solution (Beyotime, China), trans-well polycarbonate membrane cell culture inserts (Corning, Germany), Iron Assay kit (Sigma-Aldrich), T-AOC Assay and ROS Assay kit (Beyotime, China), Lyso-Tracker Red and Mito-Tracker (Beyotime, China), DPPH free radical scavenging rate detection kit (microplate method) (Leagene, China), Liperfluo and FerroOrange (DOJINDO, Japan), reduced glutathione (GSH) determination kit, hydroxyl radical (OH·) determination kit, and malondialdehyde (MDA) test kit (Nanjingjiancheng, China) were obtained.

### Cell Culture and Transfection

The human liver THLE-2 cells (Chinese Academy of Sciences, China), human hepatoma HepG2 cells (Chinese Academy of Sciences, China), HCCLM3 cells (Chinese Academy of Sciences, China), and HUH7 cells (Chinese Academy of Sciences, China) were cultured in a 37°C constant temperature incubator with 5% CO_2_ and appropriate humidity. The THLE-2 cells were cultured in BEGM containing 10% fetal bovine serum (FBS) (1% 50 U/ml penicillin). The other cells’ culture medium was DMEM containing 10% FBS (1% 50 U/ml penicillin). In subsequent experiments, when the cell density was increased to 80%, the cells were digested with 0.25% trypsin. In this study, we chose according to the purpose of the experiment.

For this study, we chose si-LC3Ⅱ and si-NCOA4 (RIBOBIO, China) with a silence effect for the purpose of the experiment. The sequences of siLC3Ⅱ and siNCOA4 are 5′-GAA​GGC​GCU​UAC​AGC​UCA​ATT-3′ and 5′-CAA​CTG​TCC​TGC​TCT​TTG​A-3′.

After logarithmic phase culture of HCCLM3 and HUH7 cells, the cells were transfected with 50 nM siRNA and the analysis was carried out according to the conventional transfection reagent protocol. FTH1 pCDNA 3.1 plasmid (Fenghuishengwu, China) with overexpression effect was selected. FTH1 plasmid and equivalent empty vector were transferred to HCCLM3 and HUH7 cells using Roche’s X-tremeGENE DNA transfection reagent.

### Experimental Animals

Female BALB/c nude mice (4–5 weeks old) were purchased from Beijing Shenghe Experimental Animal Technology Co., Ltd., and the Animal Protection and Use Committee of Changchun University of Traditional Chinese Medicine (IACUC) accepted the caryophyllene oxide animal experimental scheme against liver cancer. Just at the end of therapy, three nude mice from each group were randomly chosen and killed by humane cervical dislocation, and the rest were euthanized. Euthanasia was carried out by intraperitoneal injection of three times the dose of pentobarbital sodium (150 mg/kg) for a continuous 2–3 min. The euthanasia method was performed according to the AVMA animal euthanasia guidelines.

### CCK8 Detection and Grouping

THLE-2, HepG2, HUH7, and HCCLM3 cells were seeded at a density of 1×10^4^ cells per well in a 96-well plate. Caryophyllene oxide is prepared in different concentrations of 0, 10, 20, 40, 60, 80, 120, 140, and 160μM, and then added to each well. After 48 h of administration, 10 μl CCK-8 reagent was added to each well. After incubation at 37°C for 2 h, the OD value was determined at 450 nm by a microplate reader, and the effective concentration was selected for follow-up experiments.

HCCLM3 and HUH7 cells were divided into four groups: control group, COL group (40 μM), COM group (80 μM), and COH group (160 μM). Twenty-four hours after inoculation, the cells were treated with different concentrations of drugs; 48 h later, the cell proliferation was detected, and the cells in the control group were treated with corresponding solvents cultured normally.

### Crystal Violet Staining

HCCLM3 and HUH7 cells were placed in a 12-well plate for about 24 h and incubated with different concentrations of caryophyllene oxide for 48 h. The cells were stained with a 0.1% crystal violet solution fixed with 4% neutral formaldehyde for 10 min. After washing with PBS, the cells were observed and photographed.

### Wound Healing

HCCLM3 and HUH7 cells were placed in a 12-well plate for about 24 h and incubated with different concentrations of caryophyllene oxide for 48 h. The cells were scratched in the middle of the well with a 200-μl tip, washed three times with PBS, and then each well was added to the culture medium, placed into a 37°C, 5% CO_2_ incubator, and cultured; then, the healing degree of the scratches was measured at 0, 24, and 48 h, respectively.

### Cell Migration

The trans-well chamber was placed on a 24-well plate. HUH7 and HCCLM3 cells were resuspended in DMEM containing 0.5% FBS. In total, 2 × 10^3^ cells were inoculated into the upper chamber of the trans-well chamber at a volume of 200 μl. In the lower chamber of the trans-well chamber, 500 μl of DMEM containing 10% FBS was added and cultured for 24 h in an incubator at 37°C, 5% CO_2_, and saturated humidity. After the culture was finished, the chamber was taken out and stained with a 0.1% crystal violet solution fixed with 4% neutral formaldehyde for 5 min. After washing with PBS, the trans-well chamber was observed and photographed under a 20 × microscope. The difference in the number of cells passing through the membrane represents the change in tumor metastatic ability.

### Annexin V-FITC/PI

HCCLM3 and HUH7 cells were placed in a 12-well plate and incubated with different concentrations of caryophyllene oxide for 48 h. The culture medium was collected, washed twice with PBS, digested with trypsin without EDTA, centrifuged, and resuspended in Annexin V-binding buffer solution (1 ml), mixed with 10 μl of Annexin V-FITC/PI dye, stained for 20 min at room temperature, and flow cytometry (BD C6 PLUS) was performed.

### T-AOC Assay

HCCLM3 and HUH7 cells were placed in a six-well plate for 24 h and incubated for 48 h with different concentrations of caryophyllene oxide. About 10^6^ cells were collected without accurate counting, scraped off, placed in 200 μl cold PBS, homogenated by ultrasound to fully break the cells and release antioxidants, and centrifuged at 4°C at 12,000 g for 5 min according to the kit’s instructions; the supernatant was taken for follow-up determination, and results were calculated.

### DPPH Free Radical Scavenging

HCCLM3 and HUH7 cells were placed in a six-well plate and then incubated with different concentrations of caryophyllene oxide for 48 h. The culture medium was collected, and the cells were digested with trypsin without EDTA. Nitrogen-free radical extract of 0.8 ml was added to every 5 × 10^6^ cells, ultrasonic fragmentation was performed, and the extract was centrifuged for 10 min. The supernatant was used for follow-up determination, and the results were calculated in accordance with the kit’s instructions.

### ROS Level

HCCLM3 and HUH7 cells were placed in a 12-well plate for 24 h and incubated with different concentrations of caryophyllene oxide for 48 h. The DCFH-DA was diluted to 10 μM in the serum-free medium and incubated for 20 min at 37°C. Every 3–5 min, the mixture was turned upside down to ensure that the probe makes complete contact with the cell. The cells were washed three times with a serum-free cell culture medium. The cells were digested with trypsin without EDTA and resuspended with HBSS. Flow cytometry was used to detect the cells, and a confocal microscope was used to image them.

### Iron Assay

HCCLM3 and HUH7 cells were laid in a 12-well plate and then incubated with different concentrations of caryophyllene oxide for 48 h. The cells were digested with trypsin without EDTA, added in four times cell volume iron assay buffer for quick homogenizing, and centrifuged at 4°C for 10 min to collect the supernatant. The supernatant was collected for follow-up determination according to the kit’s instructions, and then the results were calculated.

### Detection of Lipid Peroxidation

The cells were placed in a 12-well plate and then incubated with different concentrations of caryophyllene oxide for 48 h. The cells were washed once with serum-free DMEM. Two milliliter of serum-free DMEM was used to dilute the sample. Within 30 min of incubation at 37°C, DMEM was collected, washed twice with HBSS, the cells were digested with trypsin and resuscitated cells with HBSS, detected by flow cytometry, and imaged with a confocal microscope.

### Cell FerroOrange

The cells were placed in a 12-well plate and then incubated with different concentrations of caryophyllene oxide for 48 h. Cells were washed with serum-free DMEM once. Appropriate amount of FerroOrange working solution diluted with serum-free DMEM was added to continue to culture cells for 30 min. Then, the culture medium was discarded. The cells were washed three times with HBSS and confocal microscope images were taken.

### Co-Staining of Mitochondria and Lysosomes

HCCLM3 and HUH7 cells were placed in a 12-well plate and then incubated with different concentrations of caryophyllene oxide for 48 h, and the cells were washed with PBS. Then, the Lyso-Tracker Red (LTR) probe was added to the culture medium until the final concentration reached 100 nM, followed by the Mito-Tracker Green (MTG) probe. The solution was collected, the cells were washed with PBS for three times, and the confocal microscope was used for imaging after 30 min of culture at 37°C.

### Western Blot

HCCLM3 and HUH7 cells were placed in a six-well plate with a density of 5 × 10^5^ cells per well for 24 h and then incubated with different concentrations of caryophyllene oxide for 48 h. Total protein was extracted using a total protein extraction kit (Kang Wei Century, Beijing). The protein concentration was measured using the BCA kit instructions (Beyotime, Shanghai). The proteins were collected, denatured with boiling water, and separated using SDS-PAGE at 12.5% (Kangwei Century, Beijing). The protein was transferred from the gel to the NC membrane (Milli-pore, United States). After 2 h of shaking at room temperature with 5% low-fat milk powder dissolved in TBST, the membrane was incubated overnight at 4°C with the appropriate primary antibody. The membrane and the second antibody were incubated in a shaker at room temperature for 1 h after being washed three times with 1 TBST (Servicebio, China). Finally, the expression level of each protein was determined using an ECL chromogenic kit (Thermo, United States) and the gel imaging system, using GAPDH as the internal reference. The Western blot analysis revealed the expression levels of Nrf2, HO-1, NQO1, Gpx4, NCOA4, FTH1, and LC3II.

### Immunofluorescence

HCCLM3 and HUH7 cells were placed in a 12-well plate with a density of 1 × 10^5^ cells per well for 24 h, different concentrations of caryophyllene oxide were added to incubate for 48 h, and then cells were washed with PBS. 4% paraformaldehyde for 30 min, in 4% paraformaldehyde, added to 1% BSA with 0.5% Triton, sealed at room temperature for 2 h, diluted first antibody was added, reacted overnight at 4°C, washed three times with PBS, then diluted FITC or CY3 tagged secondary antibody was added. At room temperature, it was incubated in a light and wet box for 1 h, and the protein expression was observed under a fluorescence microscope.

### Xenotransplantation Experiment

HUH7 logarithmic phase cells were suspended in 1 × 10^7^/ml cell suspension and transplanted into the right hindlimb of 5-week-old BALB/c nude mice (nude = 6). The tumor development of nude mice was monitored daily after tumor formation, and the whole process was kept aseptic. The model was effectively established after 7 days when irregular spots were felt. The tumor-bearing nude mice were randomly categorized into four groups: control (equal volume of solvent), cisplatin (5 mg/kg), sorafenib (30 mg/kg), or caryophyllene oxide (50, 100, and 200 mg/kg). Every 3 days, the tumor volume and body weight of nude mice were measured, and the survival situation was evaluated. The mice were euthanized after 14 days of therapy; the five internal organs and tumors were preserved in formalin and stained with immunohistochemical, HE and Prussian blue staining.

$$\text{Tumour}\;\text{volume}\;\left({\text{mm}}^{3}\right)\;=\;\left(\text{long}\;\text{diameter}\;\text{of}\;\text{tumour}\times \text{short}\;\text{diameter}\;{\text{of tumour}}^{2}\right)/2$$

The inhibition rate was calculated using the formula:

Tumour inhibition rate = (1 - treatment group tumour volume/control tumour volume)×100%

### Prussian Blue Staining

The tumor tissue was fixed with 4% paraformaldehyde, embedded in paraffin, sliced, made transparent with xylene, dehydrated with gradient ethanol, and made into 5-μm-thick sections. Prussian blue staining was applied to soak the surface crystal material (white sediment) for 5 min in distilled water at 37°C, followed by HE staining. Finally, the clip was sealed and the Prussian blue-stained pieces were analyzed, pictured, and evaluated under the microscope.

### HE Staining

The tumor tissue was fixed with 4% paraformaldehyde, embedded in routine paraffin, sliced, transparent in xylene, dehydrated with gradient ethanol, and made into 5-μm-thick sections. After dyeing, dehydration, transparency, sealing, and HE staining, sections were observed under the microscope for image analysis.

### Immunohistochemistry

The tumor tissue was fixed in 4% paraformaldehyde, embedded in conventional paraffin embedding, sliced, made transparent in xylene, dehydrated with gradient ethanol, cultured with 3% H_2_O_2_ methanol incubation for 15 min to remove endogenous peroxidase activity, sealed with BSA for 30 min, and incubated overnight in a wet box at 4°C. After incubation, the sections were further incubated with the second antibody at room temperature for 1 h; by then, DAB staining and re-staining with hematoxylin were performed, and the mixture was sealed with neutral glue and imaged under the microscope. To assess immunostaining, Image-ProPlus6.0 software was used to select the same yellowish brown as the standard for evaluating positive immunostaining. Each photograph was analyzed to obtain cumulative light intensity, density (IOD), and pixel tissue area (AREA). From this, the average optical density value, IOD/AREA average density, is calculated (mean density).

### Biochemical Detection

The inhibition rates of malondialdehyde (MDA), reduced glutathione (GSH), and hydroxyl radical (OH·), and the content of Fe^2+^ in serum were detected according to the instructions provided in the biochemical kit.

### Statistical Analysis

The data are presented as mean standard deviation (SD) values. At least three separate experiments’ data were statistically analyzed. GraphPadPrism6.0 program was used to perform statistical analysis or analysis of variance (ANOVA) of unpaired double-tailed Student’s test. *p < 0.05 is regarded as statistically significant.

## Results

### Inhibitory Effect of Caryophyllene Oxide on Hepatoma Cells

**FIGURE 1:**
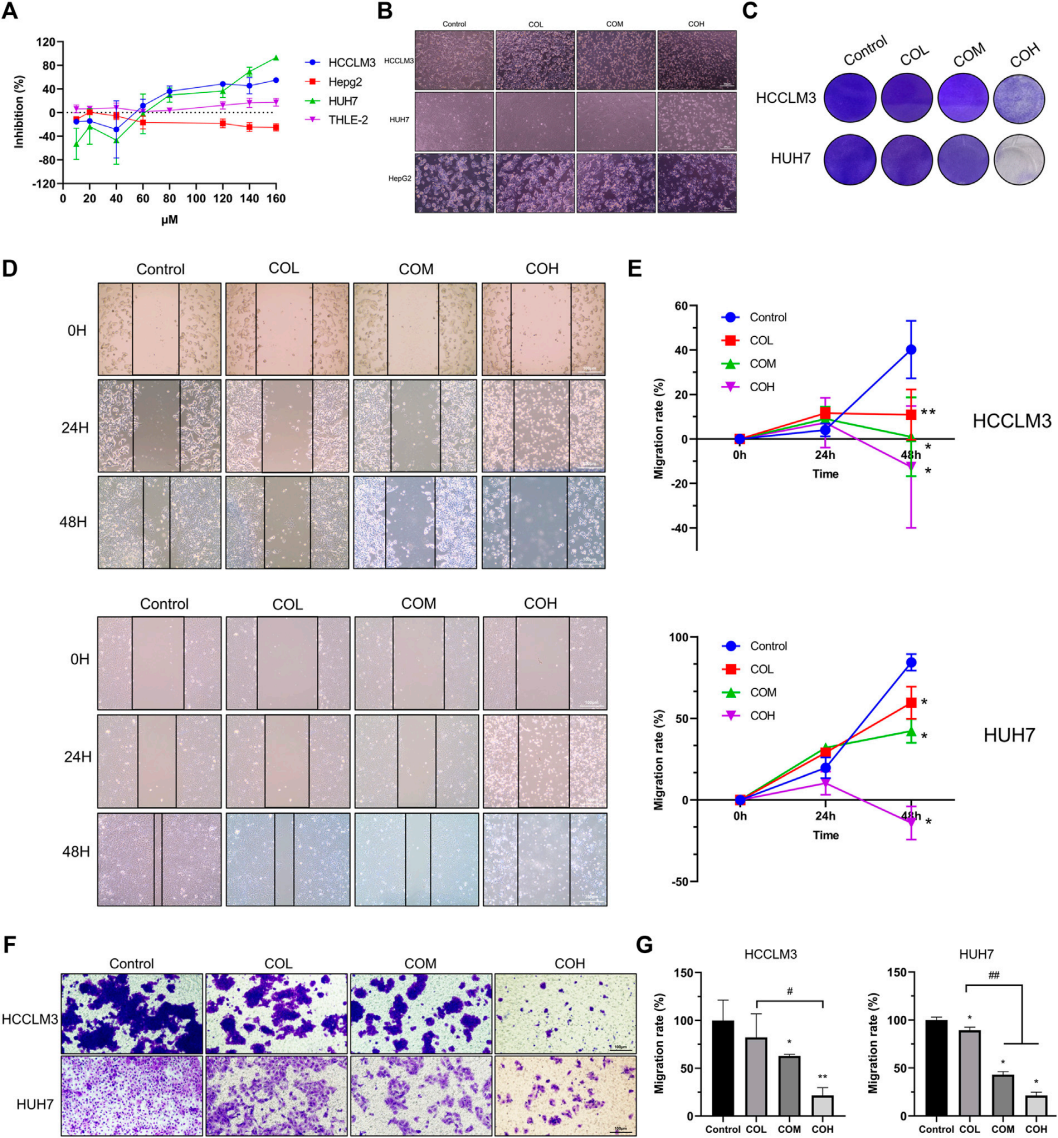
Inhibitory effect of caryophyllene oxide on HCCLM3 and HUH7 cells. **(A)** The CCK8 assay was used to determine caryophyllene oxide’s inhibition effect on the inhibition of THLE-2, HepG2, HCCLM3, and HUH7 cells after 48 h. **(B)** The morphological changes in hepatoma cells incubated with caryophyllene oxide for 48 h were observed under light microscope. **(C)** Crystal violet staining revealed the inhibition effect of caryophyllene oxide on HCCLM3 and HUH7 cells. **(D)**, **(E)** The effects of caryophyllene oxide on the migration of HCCLM3 and HUH7 cells were investigated using the cell scratch test and the trans-well experiment, and the results were presented as mobility **(F)**, **(G)**. The data were presented as mean ± SD. *p < 0.05, **p < 0.01 when compared to the control group. ^#^ *p* < 0.05, ^##^ *p* < 0.01 when compared to the COL group.

The CCK8 experiment (Figure 1A) revealed that caryophyllene oxide inhibits the proliferation of hepatoma HCCLM3 and HUH7 cells in a dose-dependent manner, but it had no obvious inhibition effects on THLE-2 and HepG2. When the concentration of caryophyllene oxide was 80μM, the inhibition rate was 27.70 ± 6.98% for HUH7 cells, 36.09 ± 5.04% for HCCLM3, and 3.74 ± 0.763% for THLE-2 cells. As a result, we chose 80 μM as the medium dose, 40 μM as the low dose, and 160 μM as the high dose for follow-up tests. There were no significant differences in the cytological morphology of HCCLM3 and HUH7 cells in the COL group. However, the cells became round or even floating in the COM group, and most cell borders in the COH group were floating, but the morphology of HepG2 cells did not significantly change after 48 h of treatment (Figure 1B). Crystal violet staining revealed a dose-dependent decrease in the number of adherent cells in the treatment group (Figure 1C). The effect of caryophyllene oxide on the migration of HCCLM3 and HUH7 cells was evaluated using cell scratch and trans-well migration assays. Caryophyllene oxide inhibited HCCLM3 and HUH7 cell migration in a dose-dependent manner (Figures 1D–G). These findings suggest that caryophyllene oxide prevents the migration of HCCLM3 and HUH7 cells.

### Effects of Caryophyllene Oxide on Apoptosis and ROS of Hepatoma Cells

**FIGURE 2:**
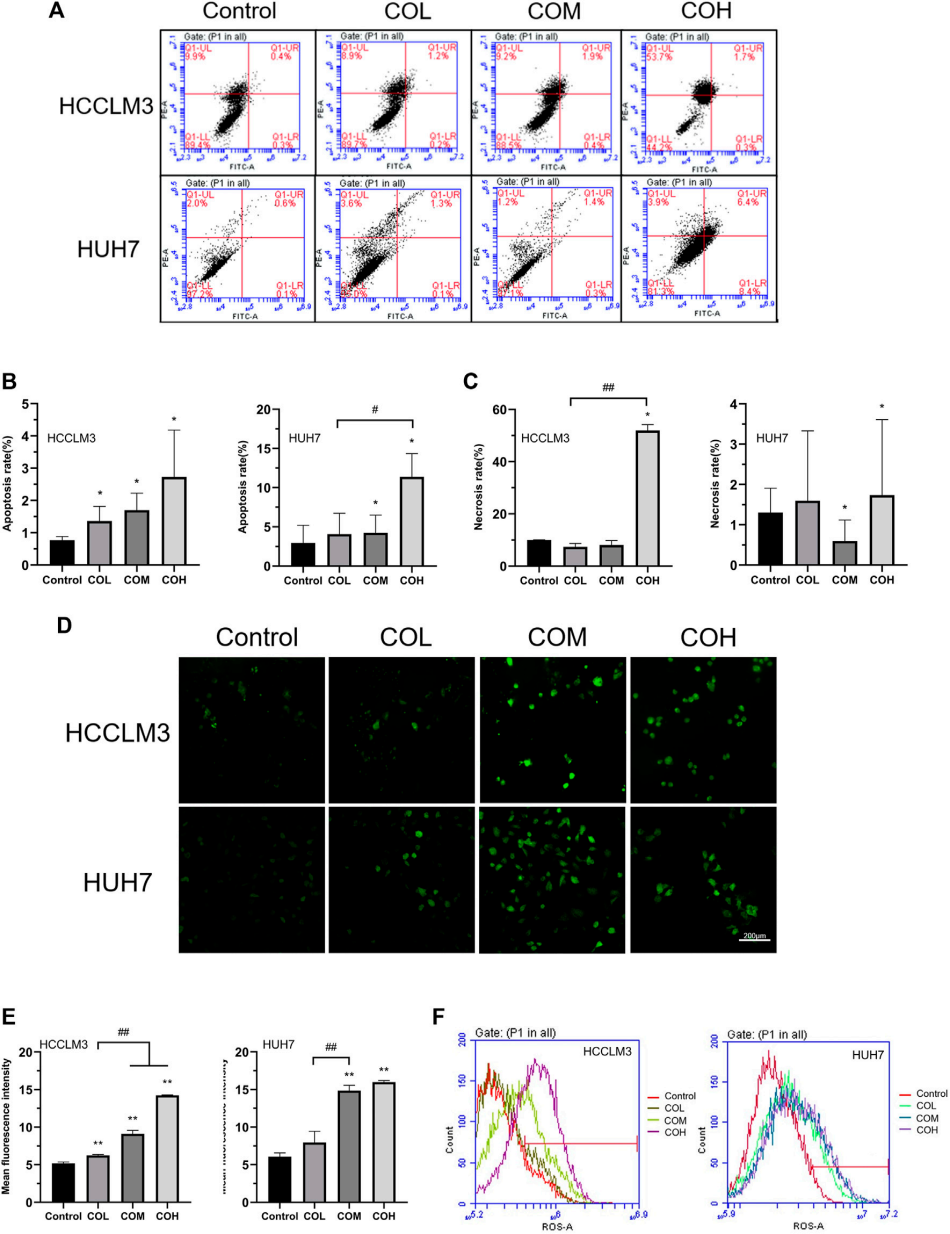
Effects of caryophyllene oxide on apoptosis and reactive oxygen species in HCCLM3 and HUH7 cells. **(A)** Flow cytometry was used to detect the Annexin V-FITC/PI of HCCLM3 and HUH7 cells, and the results were expressed as apoptosis rate and necrosis rate. **(B)**, **(C)**, **(D)**, and **(E)** Laser confocal microscopy revealed the formation of ROS in HCCLM3 and HUH7 cells (200). **(F)** Flow cytometry was used to detect ROS in HCCLM3 and HUH7 cells. The data were presented as mean ± SD. *p < 0.05, **p < 0.01 when compared to the control group. ^#^ *p* < 0.05, ^##^ *p* < 0.01 when compared to the COL group.

To explore the mechanism by which caryophyllene oxide inhibits the growth of HCCLM3 and HUH7 cells, we detected the apoptosis of the two cells in the presence of caryophyllene oxide using flow cytometry. We found that caryophyllene oxide can greatly enhance cell apoptosis and necrosis in a dose-dependent manner, after 48 h of treatment (Figures 2A–C). The apoptosis rates of HCCLM3 cells were 1.4, 2.3, and 2%, and the necrosis rates were 8.9, 9.2, and 53.7%, respectively. The apoptosis rates of HUH7 cells were 1.4, 1.7, and 14.8%, and the necrosis rates were 3.6, 1.2 and 3.9%, respectively. Meanwhile, we found that after treatment of HCCLM3 and HUH7 cells with caryophyllene oxide for 48 h, the number of reactive oxygen species (ROS) produced by the two cell lines significantly increases when assessed under fluorescence microscopy (Figures 2D,E). Similar results were obtained by flow cytometry (Figure 2F).

### Effects of Caryophyllene Oxide on Antioxidant Capacity and Protein Expression in Hepatocellular Carcinoma Cells

**FIGURE 3:**
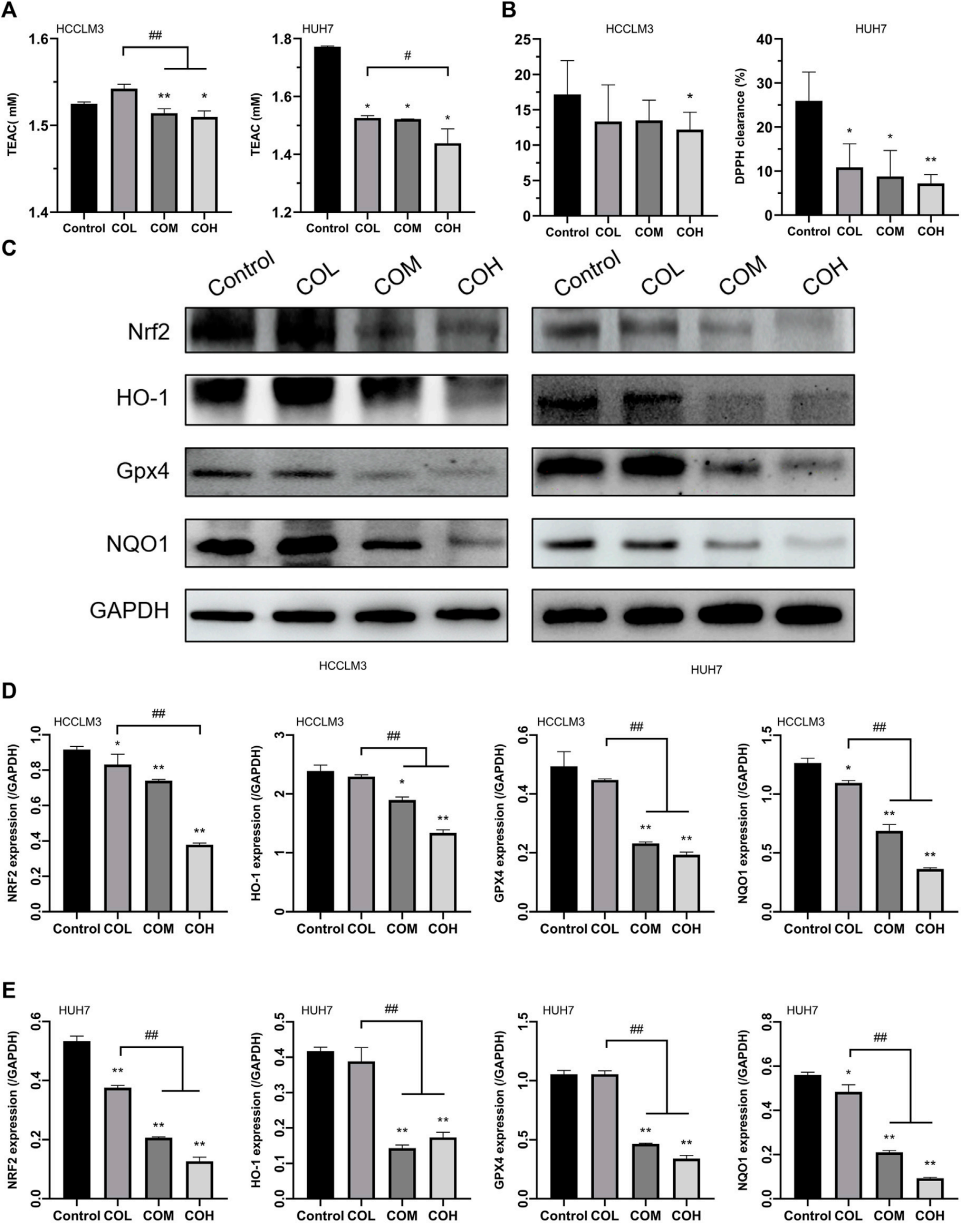
Effects of caryophyllene oxide on intracellular antioxidant capacity and antioxidant proteins in HCCLM3 and HUH7 cells. **(A)** The T-AOC assay was used to measure the antioxidant capacity of HCCLM3 and HUH7 cells. **(B)** The DPPH free radical scavenging rate in HCCLM3 and HUH7 cells. **(C)**, **(D)**, and **(E)** Western blot was used to evaluate antioxidant protein expression in HCCLM3 and HUH7 cells. The data were presented as mean ± SD. *p < 0.05, **p < 0.01 when compared to the control group. ^#^ *p* < 0.05, ^##^ *p* < 0.01 when compared to the COL group.

It was found that ROS was closely related to the level of intracellular oxidative stress (Apel and Hirt, 2004; Kattoor et al., 2017; Nakai and Tsuruta, 2021). Therefore, we evaluated the level of intracellular oxidation. Caryophyllene oxide significantly inhibited the expression of total antioxidant T-AOC in HCCLM3 and HUH7 cells (Figure 3A) and reduced the scavenging rate of DPPH free radicals (Figure 3B). Western blot showed that the expression of the antioxidant proteins NRF2, HO-1, GPX4, and NQO1 in COM and COH groups was significantly lower than that in the control group (Figures 3C–E). In conclusion, caryophyllene oxide can significantly inhibit the expression of antioxidants and antioxidant proteins in HCCLM3 and HUH7 cells.

### Effects of Caryophyllene Oxide on Free Iron and Lipid Peroxidation in Hepatocellular Carcinoma Cells

**FIGURE 4:**
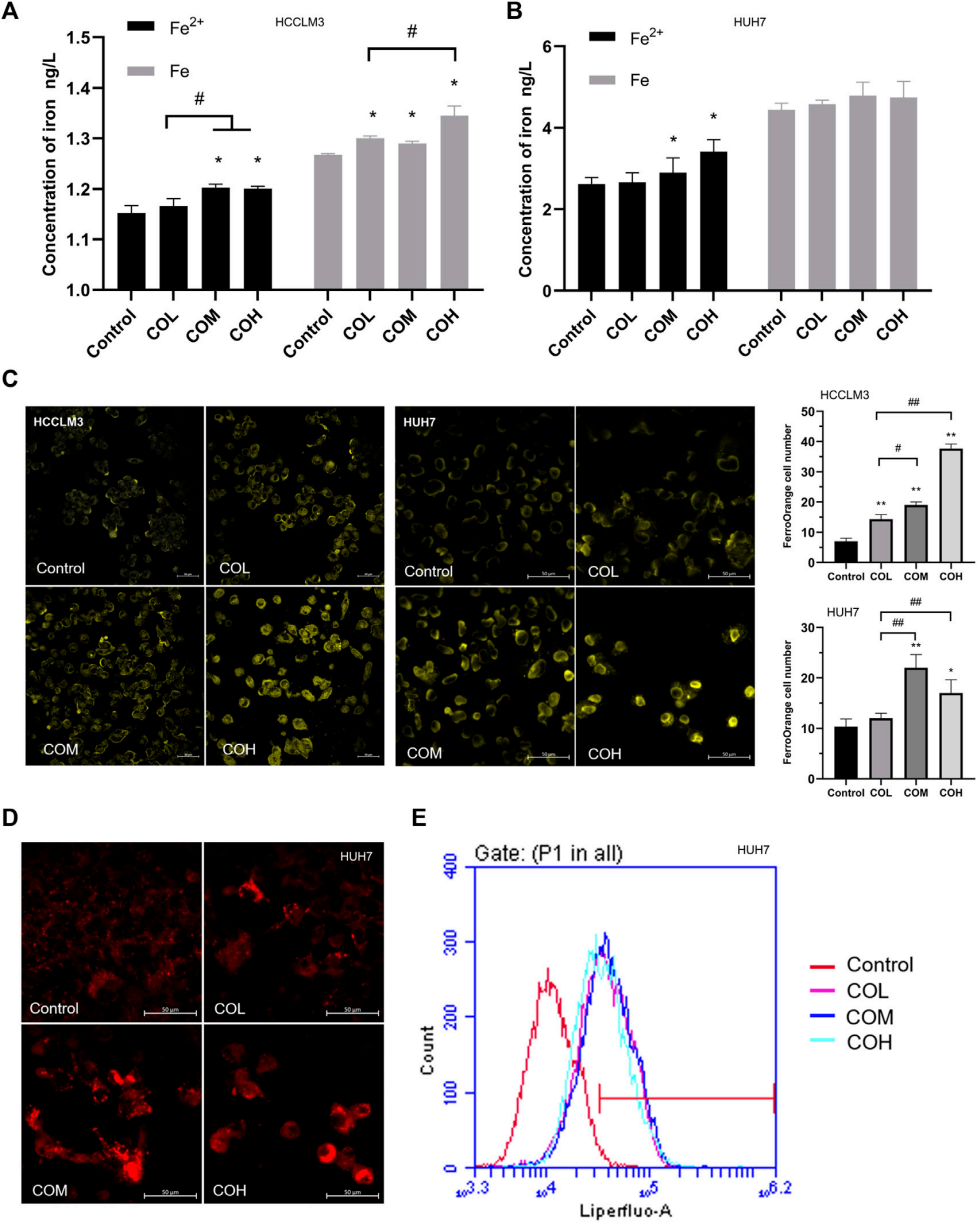
Effects of caryophyllene oxide on iron content and lipid peroxidation in HCCLM3 and HUH7 cells. **(A)**, **(B)** Iron assay kit was used to measure the levels of Fe (total iron) and Fe^2+^ in HCCLM3 and HUH7 cells. **(C)** The intracellular free iron in HCCLM3 and HUH7 cells was detected using the FerroOrange living cell probe. **(D)** Lipid peroxidation in HUH7 cells was observed using the Liperfluo living cell probe. **(E)** Flow cytometry was used to measure the level of lipid peroxidation in HUH7 cells. The data were presented as mean ± SD. *p < 0.05, **p < 0.01 when compared to the control group. ^#^ *p* < 0.05, ^##^ *p* < 0.01 when compared to the COL group.

It was reported that an increase in ROS could be triggered by an excess of Fe^2+^ (Stowe and Camara, 2009; Stockwell et al., 2017; Su et al., 2020). After 48 h incubation with caryophyllene oxide, the iron content of HCCLM3 and HUH7 cells was determined using various methods. The results of the Iron Assay Kit suggested that the content of Fe^2+^ and total iron in HCCLM3 and HUH7 cells is significantly increased (Figures 4A,B). Using FerroOrange living cell probe marking, we observed that free iron in HCCLM3 and HUH7 cells is significantly increased (Figure 4C). Various studies have shown that intracellular iron overload could lead to the formation of intracellular lipid peroxidation (Yang et al., 2020; Protchenko et al., 2021). Liperfluo living cell probe staining revealed that the level of intracellular lipid peroxidation significantly increases after incubation with caryophyllene oxide for 48 h (Figure 4D). The results of flow cytometry were similar to those observed by confocal microscopy (Figure 4E).

### Effects of Caryophyllene Oxide on Autophagy and Ferritinophagy of Hepatocellular Carcinoma Cells

**FIGURE 5:**
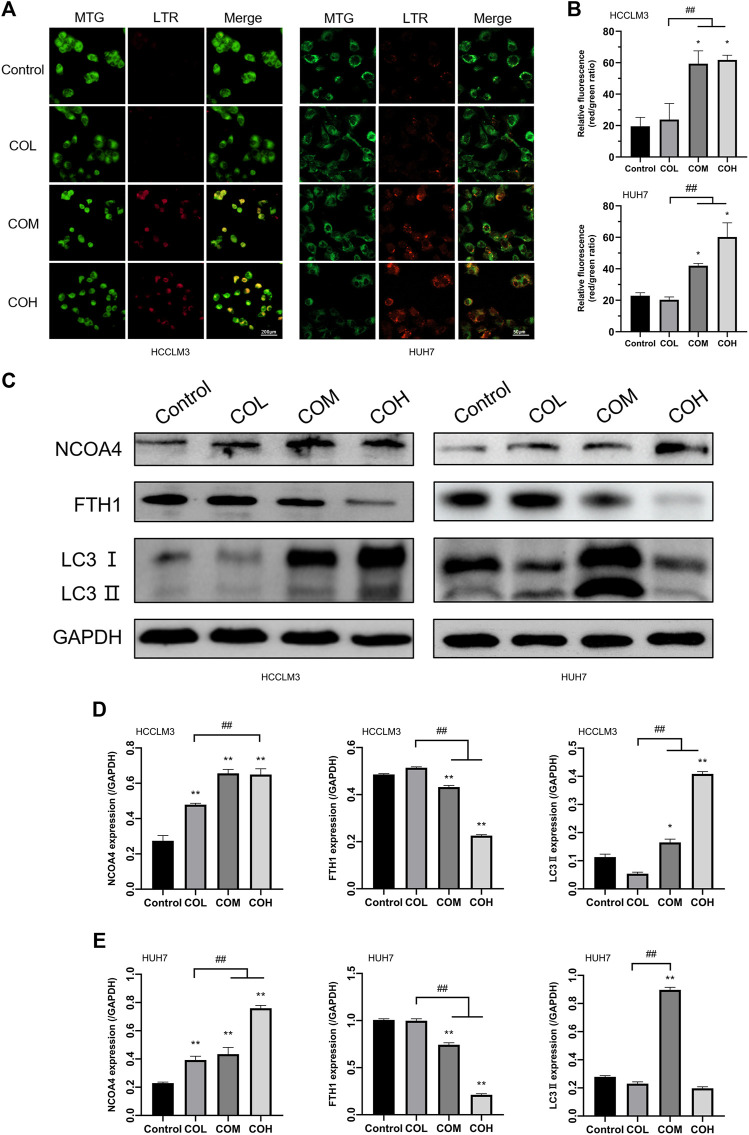
Effects of caryophyllene oxide on lysosome and ferritinophagy in HCCLM3 and HUH7 cells. **(A)** Lyso-Tracker Red and Mito-Tracker Green co-staining to investigate the autophagy–lysosome situation in HCCLM3 and HUH7 cells and expressed as **(B)**, **(C)**. Western blot analysis revealed the expression of ferritinophagy-related proteins in HCCLM3 and HUH7 cells, which were expressed as **(D)**, **(E)**. *p < 0.05, **p < 0.01 when compared to the control group. ^#^ *p* < 0.05, ^##^ *p* < 0.01 when compared to the COL group.

Intracellular iron overload occurs, which induces intracellular lipid peroxidation, resulting in altered levels of intracellular autophagy (Dodson et al., 2019; Su et al., 2019; Chen et al., 2021). The effects of caryophyllene oxide on mitochondria and lysosomes in HCCLM3 and HUH7 cells were detected by MTG and LTR co-staining. After 48 h of incubation with caryophyllene oxide, we observed that the green fluorescence of mitochondria decreased and the red fluorescence of lysosomes increased in a dose-dependent way (Figures 5A,B). These results suggest that caryophyllene oxide could induce mitochondrial damage, which is also a reason for the increase in the ROS level. At the same time, the increase in lysosomes is also a sign of the increase in the autophagy level. Western blot showed that caryophyllene oxide significantly enhanced the expression of NCOA4 and LC3II in HCCLM3 and HUH7 cells while inhibiting the expression of FTH1 (Figures 5C–E). In summary, caryophyllene oxide might induce ferritinophagy in HCCLM3 and HUH7 cells, inhibit cell growth, and promote cell death.

### Caryophyllene Oxide-Induced Ferritinophagy in Hepatoma Cells Is Dependent on NCOA4, LC3II, and FTH1 Proteins

**FIGURE 6:**
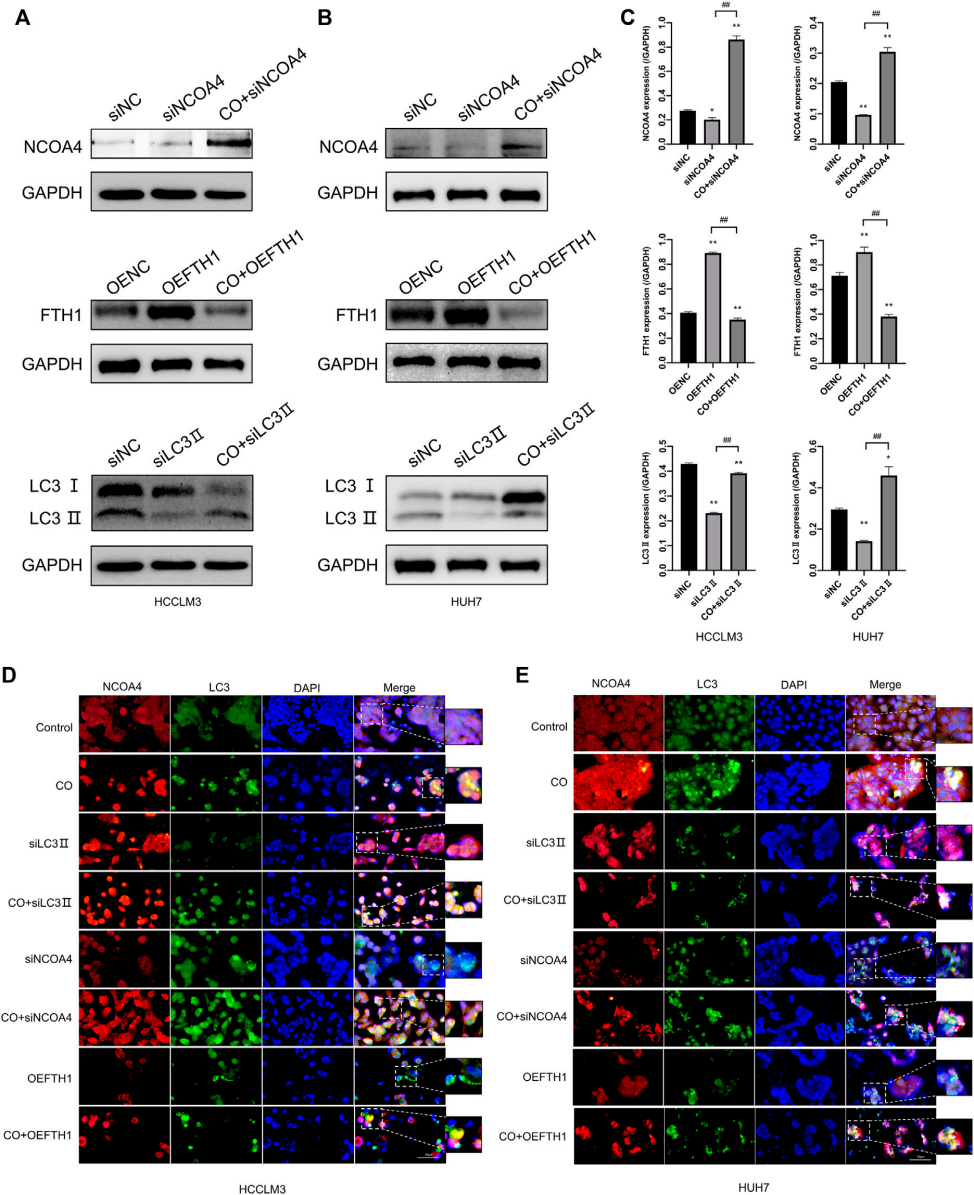
Effects of caryophyllene oxide on ferritinophagy in HCCLM3 and HUH7 cells after siLC3Ⅱ, siNCOA4 silencing, and OEFTH1 overexpression. **(A)**, **(B)**, and **(C)** Western blot identified the efficacy of siLC3Ⅱ, siNCOA4 silencing, and OEFTH1 overexpression, as well as the regulation of ferritinophagy-related proteins by caryophyllene oxide. The immunofluorescence staining **(D)**, **(E)** was used to examine the effect of caryophyllene oxide on the co-localization expression of NCOA4 and LC3II, as well as the effect of siLC3Ⅱ, siNCOA4 silence, and OEFTH1 overexpression on the co-localization expression of NCOA4 and LC3II. The data were presented as mean ± SD. *p < 0.05, **p < 0.01 when compared to the NC group. ^#^ *p* < 0.05, ^##^ *p* < 0.01 when compared to the siLC3, siNCOA4, and OEFTH1 groups.

We used immunofluorescence staining to determine the co-localization of LC3 Ⅱ and NCOA4 by fluorescence microscopy to determine whether caryophyllene oxide induces ferritinophagy (Figures 6D,E). After incubation with caryophyllene oxide (CO, 80 μM) for 48 h, many bright green spots appeared in HCCLM3 and HUH7 cells of the CO group, indicating that Lc3I was transformed into Lc3Ⅱ, leading to its transfer from the cytoplasm to autophagosomes, indicating the occurrence of autophagy. Enhanced red fluorescent spots indicated the increase in NCOA4 protein expression, while increased yellow spots indicated that LC3Ⅱ and NCOA4 co-localized and that ferritinophagy occurred (Lin et al., 2020).

To investigate if the molecular mechanism of ferritinophagy is induced by caryophyllene oxide in HCCLM3 and HUH7 cells, which would regulate the expression of NCOA4, LC3Ⅱ, and FTH1 proteins, we applied gene silencing and overexpression experiments to locate these proteins using the Western blot (Figures 6A–C) and immunofluorescence staining (Figures 6D,E). After LC3 Ⅱ silencing, the expression of LC3 Ⅱ in the siLC3Ⅱ group significantly decreased compared with that in the CO group; however, the expression of LC3Ⅱ green fluorescence was not obvious, the expression of NCOA4 with red fluorescent was not affected, and almost no yellow spots were observed, indicating that cellular autophagy was inhibited. When caryophyllene oxide and siLC3Ⅱ were associated, the expression of LC3Ⅱ protein increased significantly, as did the production of a green fluorescent LC3Ⅱ and yellow spots, indicating that caryophyllene oxide could regulate autophagy and trigger ferritinophagy *via* up-regulating the expression of LC3Ⅱ protein. Moreover, when NCOA4 was silenced (Figures 6A–C), the expression of NCOA4 protein and the expression of red fluorescent NCOA4 were significantly reduced. When caryophyllene oxide and siNCOA4 were combined, the expression of LC3Ⅱ protein, green fluorescent, and yellow spots significantly decreased compared to those in the CO group (Figure 6D). These results indicate that caryophyllene oxide promotes the occurrence of ferritinophagy by up-regulating NCOA4. When the OEFTH1 group was treated with caryophyllene oxide, the expression of FTH1 significantly decreased (Figures 6A–C). In addition, the expression of red fluorescent NCOA4 and the yellow spots were significantly reduced in the combined group compared with those in the CO group of cells (Figure 6D), which indicates that caryophyllene oxide induces ferritinophagy by increasing the expression of NCOA4 and enhancing the degradation of FTH1. Similar results were obtained with HUH7 cells. In summary, caryophyllene oxide can regulate the expression of NCOA4, LC3Ⅱ, and FTH1 proteins; trigger ferritinophagy; and induce ferroptosis in HCCLM3 and HUH7 cells.

### *In Vivo* Effects of Caryophyllene Oxide on Tumor Tissue and Iron Metabolism of Hepatocellular Carcinoma

**FIGURE 7:**
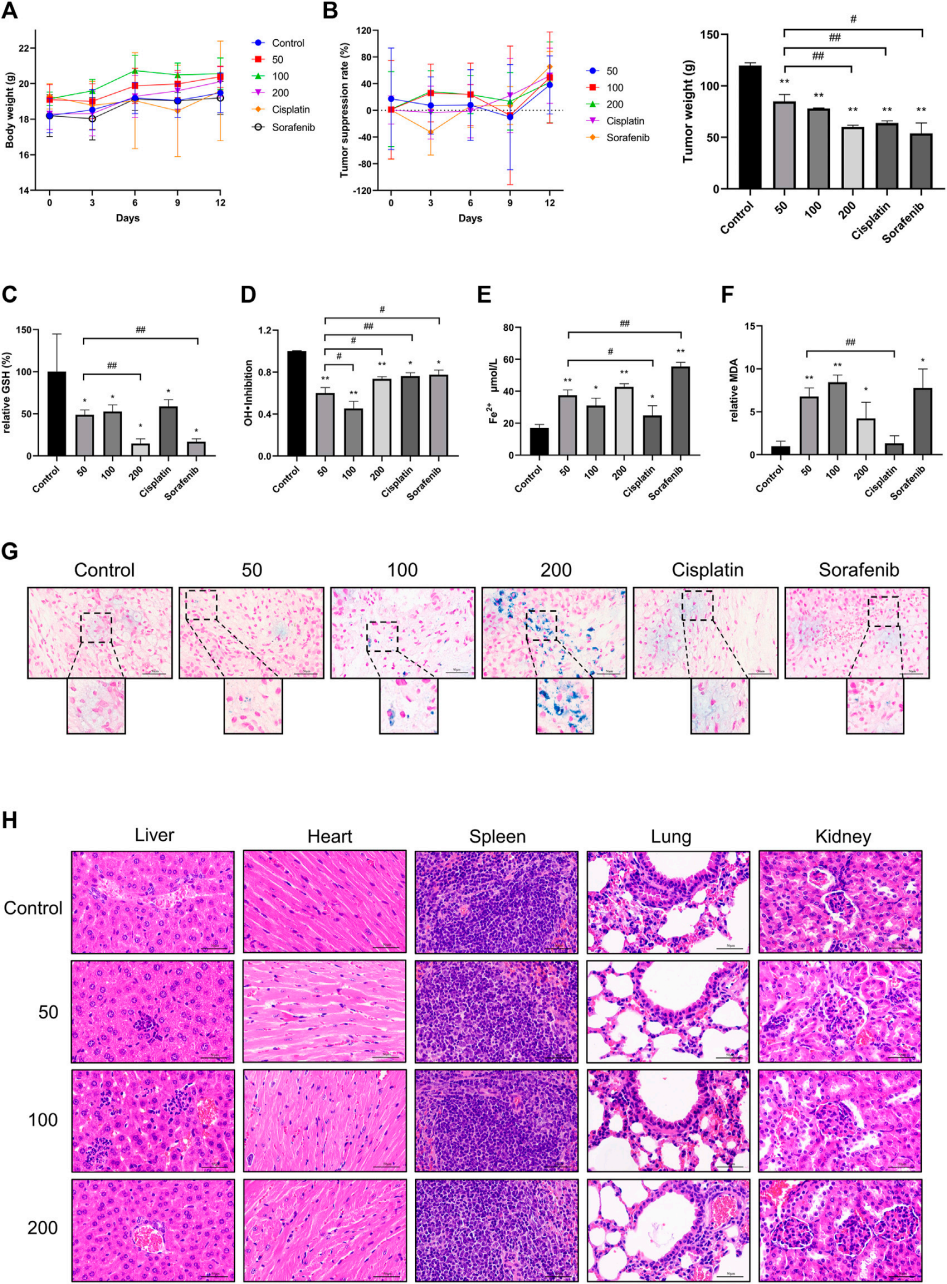
Effects of caryophyllene oxide on tumor tissue of hepatocellular carcinoma *in vivo*. **(A)** The body weight of mice was measured every 3 days, **(B)** and the length and width of the transplanted tumor were measured five times with a Vernier caliper. The average tumor inhibition rate was calculated as follows: (1-tumor volume of the treatment group/control tumor volume) × 100%. At the end of the experiment, serum was collected to test the inhibitory ability of **(C)** GSH and **(D)** hydroxyl radical inhibition and **(E)** Fe^2+^ and **(F)** MDA lipid peroxidation. **(G)** Prussian blue staining was used to detect ferritinophagy in tumor tissues. **(H)** HE staining was applied to detect pathological alterations in the liver, heart, spleen, lung, and kidney of nude mice produced by caryophyllene oxide. The ruler has a scale of 50 μm. The data represented three different experiments (n = 3). The data were presented as mean ± SD. *p < 0.05, **p < 0.01 when compared to the control group. ^#^ *p* < 0.05, ^##^ *p* < 0.01 when compared to the 50 mg/kg group.

Nude mice were used to provide an animal model for HUH7 cell subcutaneous transplantation. The results showed that the body weight of nude mice, treated with three different concentrations of caryophyllene oxide (50 mg/kg, 100 mg/kg, and 200 mg/kg), is significantly increased compared with that of the control group. However, the body weight of nude mice in the cisplatin and sorafenib groups was not significantly affected (Figure 7A). The tumor inhibition rate of the caryophyllene oxide-treated groups (50 mg/kg, 100 mg/kg, and 200 mg/kg) was significantly higher than that of the control group, suggesting that caryophyllene oxide can also suppress the proliferation and induce death of HUH7 cells *in vivo* (Figure 7B).

The results of biochemical examination of the nude mice serum indicators related to iron death showed that the caryophyllene oxide groups (50 mg/kg, 100 mg/kg, and 200 mg/kg) had a significant reduction of the serum antioxidant GSH (Figure 7C) and inhibition of hydroxyl radical inhibition capacity when compared with those in the control group (Figure 7D; *p* < 0.05 or *p* < 0.01). A significantly increase in serum Fe^2+^ (Figure 7E) and MDA expression were also observed (Figure 7F) (*p* < 0.05 or *p* < 0.01). These data demonstrate that caryophyllene oxide can inhibit the expression and antioxidant capacity of antioxidants in the serum of nude mice. In addition, Prussian blue staining of the tumor tissue section was detected under microscopy. A significant increase in blue iron deposition was observed in the sections of the caryophyllene oxide-treated tumors (50 mg/kg, 100 mg/kg, and 200 mg/kg; Figure 7G), demonstrating that caryophyllene oxide may promote iron overload and deposition in, resulting in lipid peroxidation in tumor tissue.

H&E staining was used to determine if caryophyllene oxide causes pathological changes in internal organs. No significant pathological alterations in the liver, heart, spleen, lung, and kidney were observed in caryophyllene oxide-treated groups (50 mg/kg, 100 mg/kg, and 200 mg/kg) compared with those in the control group (Figure 7H).

**FIGURE 8:**
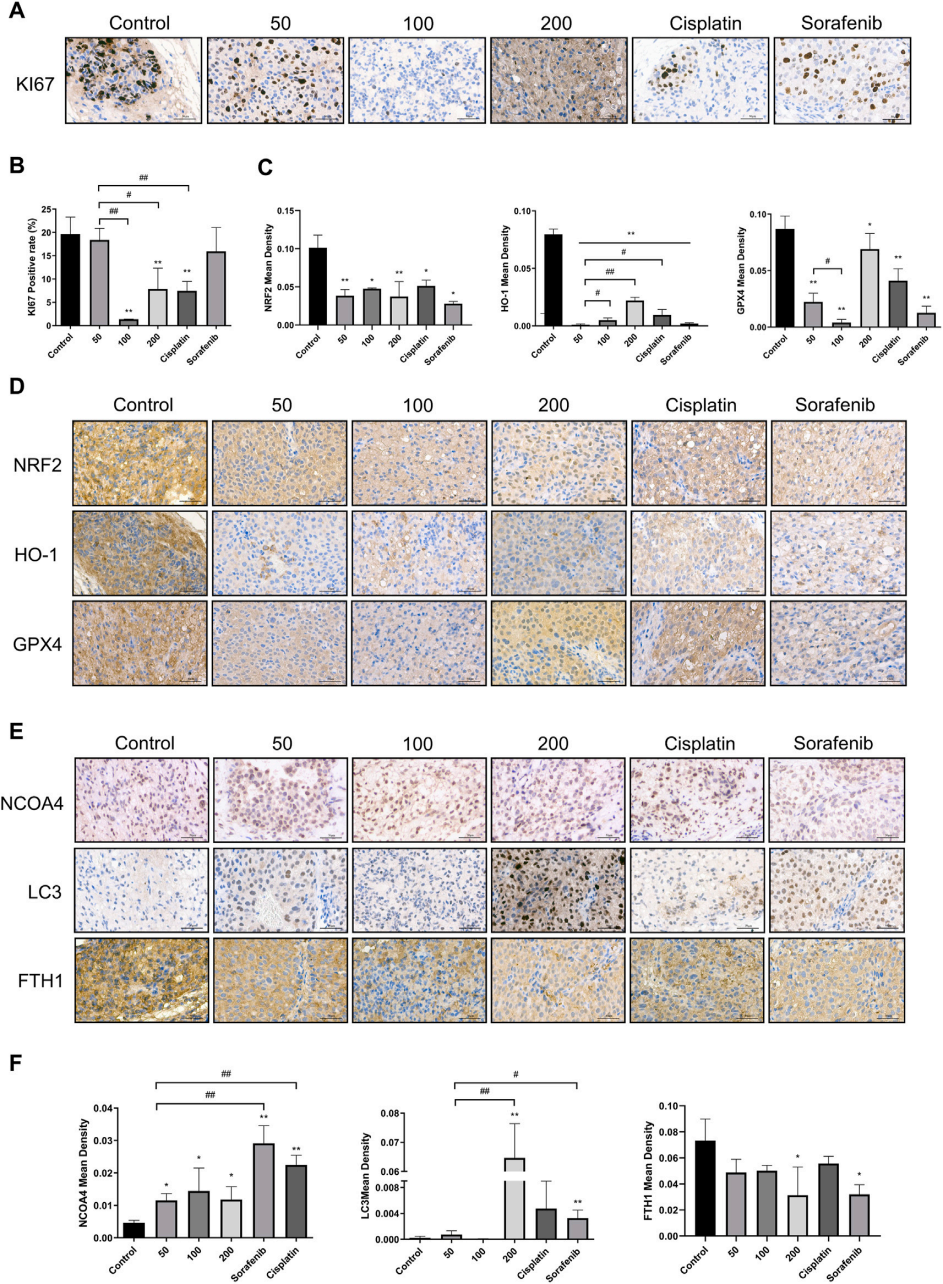
Effects of caryophyllene oxide on protein expression in hepatocellular carcinoma *in vivo*. **(A)**, **(B)** Immunohistochemistry was used to identify the expression of KI67 by caryophyllene oxide; **(C)**, **(D)** to detect the expression of NRF2, HO-1, and GPX4 protein, and **(E)**, **(F)** to detect the expression of NCOA4, LC3, and FTH1. The ruler measures 50 μm. The data were from three different tests (*n* = 3). The data were presented as mean ± SD. *p < 0.05, **p < 0.01 when compared to the control group. ^#^ *p* < 0.05, ^##^ *p* < 0.01 when compared to the 50 mg/kg group.

Immunohistochemistry was used to investigate the effect of caryophyllene oxide on the expression of oxidation and ferritinophagy-related proteins in tumor tissue. The results suggested that the positive rate of KI67 was significantly lower in the caryophyllene oxide group (100 mg/kg and 200 mg/kg) than that in the control group (Figures 8A,B; *p* < 0.05 or *p* < 0.01), indicating that caryophyllene oxide inhibits tumor growth. The expressions of NRF2, HO-1, and GPX4 were significantly lower in the caryophyllene oxide group (50 mg/kg, 100 mg/kg, and 200 mg/kg) than those in the control group (Figures 8C,D; *p* < 0.05 or *p* < 0.01), indicating that caryophyllene oxide suppresses the expression of antioxidant proteins in tumor tissue and reduces the degree of anti-oxidation. Significant increases in the expressions of LC3Ⅱ and NCOA4 and decreases in FTH1 proteins were observed in the caryophyllene oxide groups (50 mg/kg, 100 mg/kg, and 200 mg/kg) compared with those in the control group (Figures 8E,F; *p* < 0.05 or *p* < 0.01). Therefore, caryophyllene oxide can regulate the expression of ferritinophagy in cells. In summary, caryophyllene oxide suppressed anti-oxidation in nude mice tumor tissues, inhibited ferritin expression, promoted ferritinophagy-related protein expression, triggered ferritinophagy, and induced ferroptosis in tumor cells.

## Discussion

The results of iron overload associated with ferritinophagy in tumor cells showed that this process increases tumor cell ferroptosis (Tang et al., 2018; Liu et al., 2020; Ajoolabady et al., 2021). Caryophyllene oxide is a small molecular compound that can be found in various plants (Sanchez-Munoz et al., 2012; Monzote et al., 2018; Ibrahim et al., 2019). Recent studies indicated that it has an inhibitory effect on prostate cancer cells (Delgado et al., 2021), and previous research linked its antitumoral mechanism to apoptosis and mitochondrial depolarization (Delgado et al., 2021). However, the effect of caryophyllene oxide on ferritinophagy in hepatocellular carcinoma has never been reported and the precise mechanism remains unknown.

**FIGURE 9:**
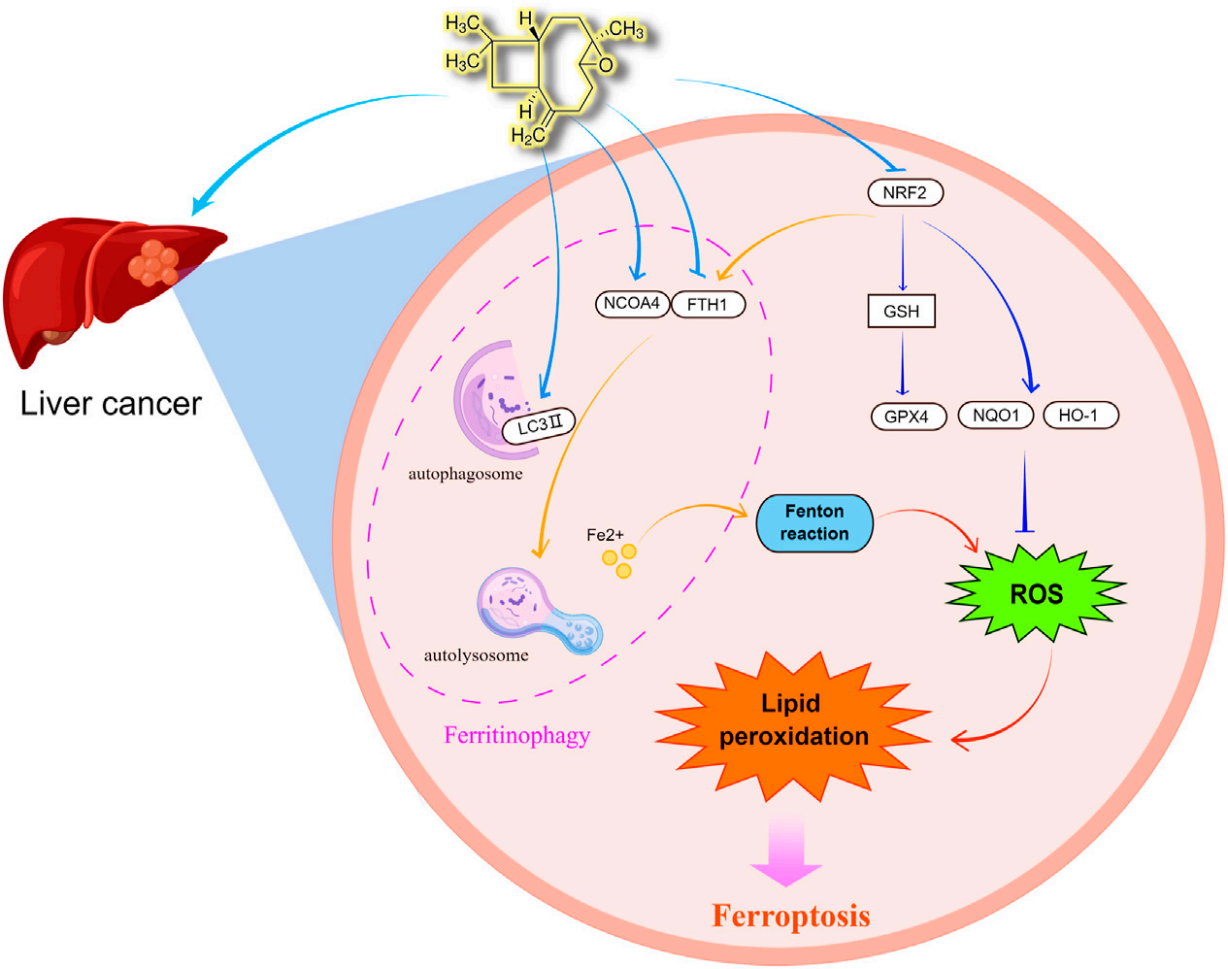
Caryophyllene oxide can regulate the expression of NCOA4 and FTH1, increase the level of autophagy regulated by lysosome and LC3 II, and then degrade ferritin to the release of free iron, consequently triggering ferritinophagy. When a cell accumulates a large amount of free iron, the Fenton reaction produces hydroxyl radical (OH·) that increases the formation of ROS and causes oxidative stress. When a large amount of ROS accumulates in cells, it can lead to the accumulation of lipid peroxidation and induce cell ferroptosis. Simultaneously, caryophyllene oxide can inhibit GSH expression by inhibiting the NRF2 signal pathway, therefore inhibit the expression of its target protein GPX4, which is the star regulator protein of ferroptosis, as well as the expression of target proteins HO-1, FTH1, and NQO1, enhance the accumulation of ROS, and finally induce ferroptosis. By Figdraw (www.figdraw.com).

In this research, we found that caryophyllene oxide inhibits the proliferation of two types of hepatocellular carcinoma cell lines: HCCLM3 and HUH7. Caryophyllene oxide also influenced their cell morphology, affected their cell migration, and increased cell apoptosis and death. According to the findings of several studies (Hou et al., 2016; Feng et al., 2020; Fuhrmann et al., 2020; Yang et al., 2021), the occurrence of ferroptosis is closely related to oxidative stress. Ferroptosis is characterized by the buildup of lipid peroxidation to deadly levels. Ferritinophagy regulates ferroptosis by releasing Fe^2+^, which increases intracellular free iron levels and activates the Fenton reaction, resulting in high levels of reactive oxygen species, which enhances oxidative stress and reduces glutathione levels, thus promoting ferroptosis. Experimentally (Figure 9), we observed that caryophyllene oxide increases the formation of ROS in HCCLM3 and HUH7 cells in a dose-dependent manner, and inhibits the scavenging of intracellular free radicals and the production of antioxidants. It has been found that NRF2 can affect cell ferroptosis by regulating HO-1, GPX4, NQO1, and FTH1, which play important roles in the protection of hepatocellular carcinoma (HCC) cells from ferroptosis (Sun et al., 2016). In this experiment, caryophyllene oxide could decrease the expression of NRF2 protein *in vivo* and *in vitro*, thus inhibiting the expression of HO-1, GPX4, and NQO1; intracellular antioxidant levels; and the expression of GSH in serum and scavenge hydroxyl radicals, which influence the oxidation level in tumor tissues. We also found that caryophyllene oxide regulates the level of intracellular Fe^2+^ and increases the content of free iron, resulting in intracellular lipid peroxidation and MDA which cause cell damage *in vivo* and *in vitro*. NCOA4 has been found to directly interact with FTH1 through its C-terminal domain and certain key residues. NCOA4 binds to the microtubule-associated protein 1 light chain 3′-phosphatidylethanolamine (LC3-PE) on the developing autophagy membrane and targets the iron-containing ferritin complex for autolysis, which is the main process of ferroptosis (Kaur and Debnath, 2015; Mancias et al., 2015). Ferroptosis can be inhibited by suppressing autophagy or decreasing NCOA4 expression, and therefore, this process can be used to develop a strategy for preventing ferroptosis (Gao et al., 2016). Furthermore, we discovered that caryophyllene oxide raises the number of intracellular lysosomes, increases the expression of NCOA4 and LC3 II, and decreases the expression of FTH1 *in vivo* and *in vitro*. Gene silencing and overexpression experiments were used to further confirm whether caryophyllene oxide was related to ferritinophagy. The results revealed that caryophyllene oxide can promote the expression of NCOA4 and LC3II after silencing, and reduce the expression of FTH1 after overexpression. We also found that caryophyllene oxide can promote the co-localized expressions of NCOA4 and LC3II, resulting in ferritinophagy. In conclusion, caryophyllene oxide can trigger ferritinophagy, increase intracellular free iron level, and promote ferroptosis in cells. According to our experimental results, caryophyllene oxide can induce ferritinophagy in tumor cells by regulating the NCOA4/FTH1/LC3II signaling pathway, which leads to ferroptosis and the inhibition of the growth of the liver cancer cells, HCCLM3 and HUH7.

Even though we conducted thorough tests to show that caryophyllene oxide promotes ferritinophagy and ferroptosis in hepatoma HCCLM3 and HUH7 cells, there are still limitations to our research. In this study, the used dose of caryophyllene oxide was relatively high. To further investigate the antitumoral efficacy of caryophyllene oxide, future experiments should adjust the dose and include other types of tumors. In summary, we believe that caryophyllene oxide, which is a small molecular compound existing in a variety of plants, is expected to be used as a drug for the treatment of liver cancer.

## Data Availability

The original contributions presented in the study are included in the article/Supplementary Material; further inquiries can be directed to the corresponding authors.
